# Effect of vitiligo treatment by compound Glycyrrhizin combined with fractional laser and Triamcinolone Acetonide injection on T Lymphocyte subpopulation

**DOI:** 10.12669/pjms.38.1.4412

**Published:** 2022

**Authors:** Ling Li, Lei Gao, Yifan Zhao

**Affiliations:** 1 Ling Li, Department of Dermatology, Baoding First Central Hospital, Baoding, Hebei 071000, China; 2 Lei Gao, Department of Dermatology, Baoding No.1 Hospital, Baoding, Hebei 071000, China; 3 Yifan Zhao, Department of Dermatology, Baoding First Central Hospital, Baoding, Hebei 071000, China

**Keywords:** Vitiligo, Compound glycyrrhizin, Fractional laser, T lymphocyte subpopulation

## Abstract

**Objectives:**

To discuss the effective mechanism of vitiligo treatment by compound glycyrrhizin combined with fractional laser and triamcinolone acetonide injection.

**Methods:**

Forty-two patients with vitiligo vulgaris in the stable phase were classified into combined group (19 cases) and medicine group (23) admitted in dermatology department, Baoding First Central Hospital from January 2017 to July 2018. Both groups took 50mg compound glycyrrhizin orally three times per day, and applied halometasone cream externally once per day. Based on this treatment method, after the combined group adopted fractional laser, triamcinolone acetonide injection encapsulation was used immediately. After the treatment for six months, the curative effect was judged for both groups. Flow cytometry was used to test the changes of T lymphocyte subpopulation in peripheral blood before and after treatment. Meanwhile, immunohistochemical method was adopted to determine CD_4_^+^ and CD_8_^+^ T lymphocyte expression level. Besides, the normal control group was set up.

**Results:**

The efficacy of combined group and medicine group were 73.68% and 56.52% respectively, *P*<0.05. The comparison of CD_3_^+^, CD_4_^+^, CD_8_^+^ and CD_4_^+^/CD_8_^+^ T lymphocyte level in serum and skin damage before and after treatment had no statistical significance (*P*>0.05). Serum CD_4_^+^ T cells of vitiligo patients reduced, compared with the normal control group (*P*<0.05), and CD_4_^+^/CD_8_^+^ declined (*P*<0.05). CD_4_^+^ and CD_8_^+^ T lymphocytes at the skin damage of patients increased, compared with normal control group (*P*<0.05).

**Conclusions:**

Compound glycyrrhizin combined with fractional laser and triamcinolone acetonide injection has good clinical effect in the treatment of vitiligo vulgaris in the stable phase, and its effective mechanism may have nothing to do with T lymphocyte subpopulation.

## INTRODUCTION

Vitiligo is a primary local or extensive skin mucosa depigmentation skin disease. The average morbidity in the world is 0.5-1.0%^1^, with racial difference. The darker the skin color, the higher the morbidity. Generally, adolescents show a high morbidity.^2^ Since this disease affects appearance, it often brings about psychological pressure to patients and affects their normal social life.^3^-^4^ The pathogenesis of vitiligo is still unclear, and there is no specific therapy. We applied compound glycyrrhizin combined with fractional laser and triamcinolone acetonide injection to treat vitiligo, and gained the good effect. We used compound glycyrrhizin combined with dot-matrix laser and triamcinolone acetonide injection to treat vitiligo and achieved good results, and discussed its effective mechanism, which is reported as follows.

## METHODS

Forty-two patients suffering from vitiligo vulgaris in the stable phase were included in the study which was conducted in the dermatology department, Baoding First Central Hospital were from January 2017 to July 2018.

### Ethical approval:

The study was approved by the Institutional Ethics Committee of Baoding First Central Hospital (No.: [2019]-001; date: 11 June, 2019), and written informed consent was obtained from all participants.

### Inclusion criteria:

1. Conformity to diagnosis standard of vitiligo vulgaris, no development of rash within six months;
2. Zood compliance, willing to receive examination and subsequent visit at a regular interval;
3. No systematic or local use of immunosuppressor within three months.

### Exclusion criteria:

1. Patients with severe hepatic and renal function incompetence, hypertension, diabetes and heart disease;
2. Women in gravidity and lactation period;
3. Patients with skin cancer and photaesthesia. The patients selected were classified into medicine group (23 cases) and combined group at random (19 cases).

Compound glycyrrhizin was applied for the medicine group, while compound glycyrrhizin combined with CO_2_ fractional laser and triamcinolone acetonide injection was applied for the combined group. There were 13 male patients and 10 female patients in the medicine group, with the age of 3~68 and average age of 35.14±15.5. The course of disease was 1~360 months, and the average course of disease was (72.95±76.34) months. There were 10 male patients and nine female patients in the combined group, with the age of 6~55 and average age of 34.05±12.45. The course of disease was 2~360 months, and the average course of disease was (76.26±67.23) months. Clinical data comparison of both groups in gender, age and course of disease had no statistical significance (*p*>0.05). Pathologic biopsy was completed for 10 patients in the medicine group before and after treatment, while hat was completed for 11 patients in the combined group. 11 patients with normal skin damage in the control group were chosen. The comparison of patients and the control group in gender, age and part had no statistical significance (*p*>0.05).

Both groups took 50mg compound glycyrrhizin tablet orally three times per day (trade name: Meineng, produced by Minophagen Pharmaceutical Co., Ltd. and sold by Shenzhen Jian’an Medical Company), and proper amount of halometasone cream (trade name: Aoneng, produced by Hong Kong Bright Future Pharmaceutical Co., Ltd.) was used externally once per day. On this basis, the combined groupwas treated with CO_2_ fractional laser (produced by Jilin King Laser Technology Co., Ltd.). The energy density was 20~40mJ/pulse, and the coverage was 10~20%. After treatment, triamcinolone acetonide injection (trade name: Transton, produced by Kuning Jida Pharmaceutical Co., Ltd.) was encapsulated for ten minutes immediately once per three weeks. The curative effect was observed after 6 months. After the course of treatment ended, follow-up visit continued, and adverse effects and recurrence rate were evaluated.

### Observation Indicators:

(1) Clinical effect criteria^5^: healed: vitiligo fades away completely; significantly effective: vitiligo area decreases or fades away, and the area of normal skin color recovered is 50% or above; improved: vitiligo fades away or decreases; ineffective: no pigment of vitiligo regenerates or the scope expands. Effective rate = cure rate + significantly effective rate. (2) Adverse effects: adverse effects of both groups were observed. (3) Peripheral blood T lymphocyte subpopulation expression: 2ml peripheral fasting venous blood was gathered for both groups, and flow cytometry (produced by American Beckmann Corporation, model: Epics XL) was used to detect CD_3_^+^, CD_4_^+^ , CD_8_^+^ and CD_4_^+^/CD_8_^+^ T lymphocyte level. (4)CD_4_ and CD_8_ T lymphocyte immunohistochemistry was conducted for skin damage tissues.

### Statistical Analysis:

SPSS13.0 statistics software was used for analysis. Measurement data were expressed with x̄±s, and independent t test was used for intra-group comparison. Enumeration data were expressed with frequency and percentage, and χ^2^ test was adopted for inter-group comparison. P<0.05 means differences have statistical significance.

## RESULTS

Three patients cured; eleven had significant effect; four improved; one had no effect; the effective rate was 73.68%. In the medicine group, two patients cured; eleven had significant effect; seven improved; three had no effect; the effective rate was 56.52%. The effect rate of combined group was obviously higher than that of medicine group (*P*<0.05).

**Fig.1:**
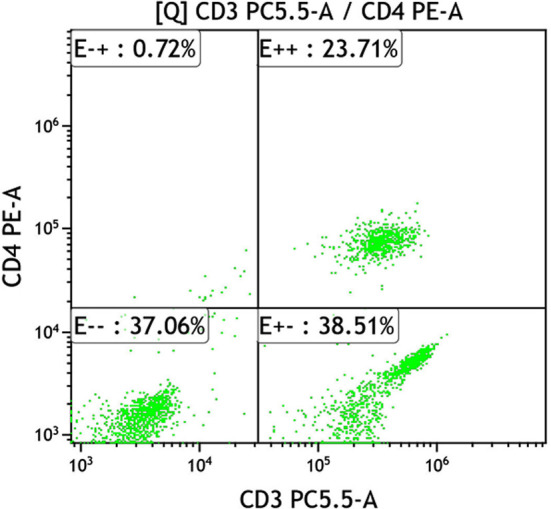
CD_4_^+^ T lymphocyte expression of combined group before treatment.

**Fig.2:**
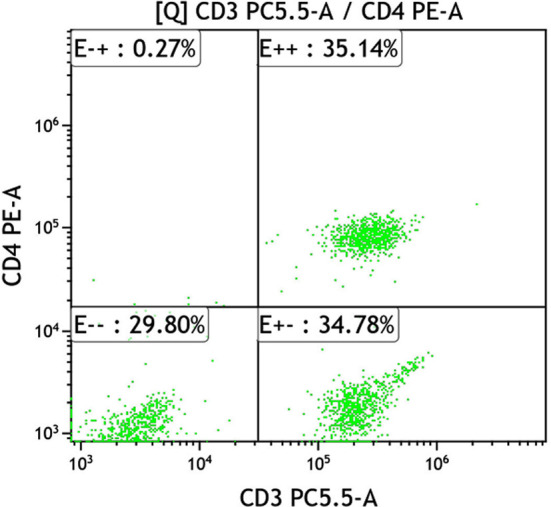
CD_4_^+^ T lymphocyte expression of combined group after treatment.

**Fig.3:**
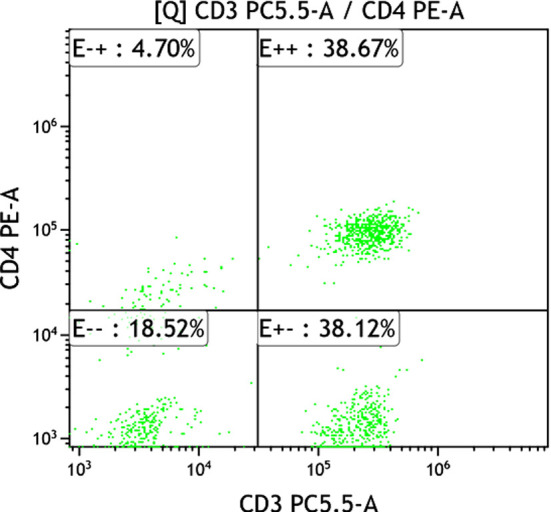
CD_4_^+^ T lymphocyte expression of normal control group.

Three patients felt causalgia, and was relieved after the treatment ended, without special treatment; two showed facial edema, was relieved spontaneously after drug withdrawal and quitted the experiment. (Figure.1-3) Comparison of T lymphocyte subpopulation expression level of both groups before and after treatment is shown in Table-I. After treatment, CD_3_^+^ and CD_4_^+^ T lymphocyte of both groups slightly increased; CD_8_^+^ T lymphocyte slightly declined; CD_4_^+^/CD_8_^+^ did not rise significantly; the comparison before and after treatment had no statistical significance (*p*>0.05). T lymphocyte subpopulation of both groups had no obvious change before and after treatment. The comparison between two groups of vitiligo patients before treatment and normal control group is shown in Table-II. Both CD_4_^+^ T lymphocyte and CD_4_^+^/CD_8_^+^ of vitiligo patients declined, compared with the normal control group (*P*<0.05). The comparison of CD_3_^+^ and CD_8_^+^ T lymphocyte had no statistical significance (*P*>0.05).

**Table I:** Comparison of T lymphocyte subpopulation expression level of combined group and medicine group before and after treatment (x̅ ±s).

Group	n	CD_3_^+^(%)	CD_4_^+^(%)	CD_8_^+^(%)	CD_4_^+^/CD_8_^+^
Combined group	19				
Before treatment		53.64±9.84	32.02±7.98	22.85±4.10	1.41±0.29
Treatment for 6 months		55.96±8.97	33.12±7.39	21.94±2.64	1.51±0.27
p		0.452	0.662	0.422	0.278
Medicine group	23				
Before treatment		52.79±9.82	31.64±8.02	20.80±3.19	1.54±0.43
Treatment for 6 months		53.68±9.53	34.77±8.71	20.63±2.51	1.70±0.42
p		0.755	0.211	0.841	0.218

***Note:*** comparison of both groups before and after treatment, P>0.05.

**Table II:** Comparison of T lymphocyte subpopulation expression level of vitiligo patients and normal control group (x̅ ±s).

Item	n	CD_3_^+^(%)	CD_4_^+^(%)	CD_8_^+^(%)	CD_4_^+^/CD_8_^+^
Vitiligo patients	42	53.17±9.72	31.81±7.91	21.72±3.73	1.48±0.38
Control group	23	57.33±10.11	36.90±9.22	20.43±2.76	1.82±0.47
p		0.115	0.031	0.117	0.004

***Note:*** CD_4_^+^ and CD_4_^+^/CD_8_^+^ Comparison between vitiligo patients and normal control group, P<0.05.

**Fig.4:**
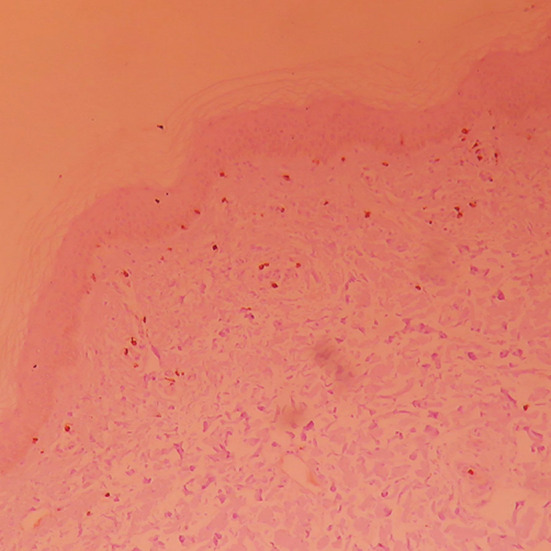
CD_4_^+^ lymphocyte expression in skin damage tissue of normal control group (×100).

**Fig.5:**
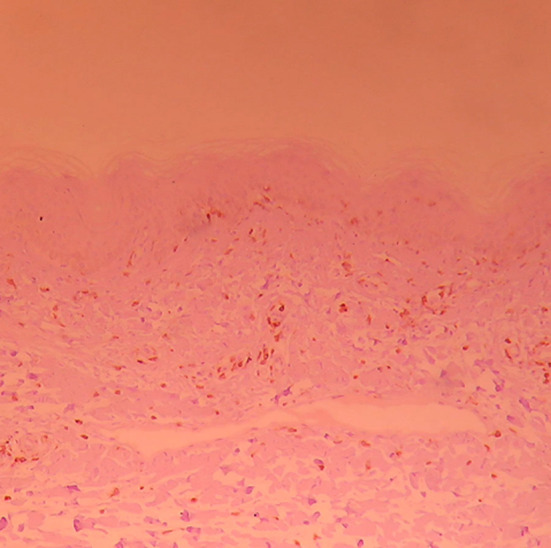
CD_4_^+^T lymphocyte expression in skin damage tissue before combined treatment (×100).

**Fig.6:**
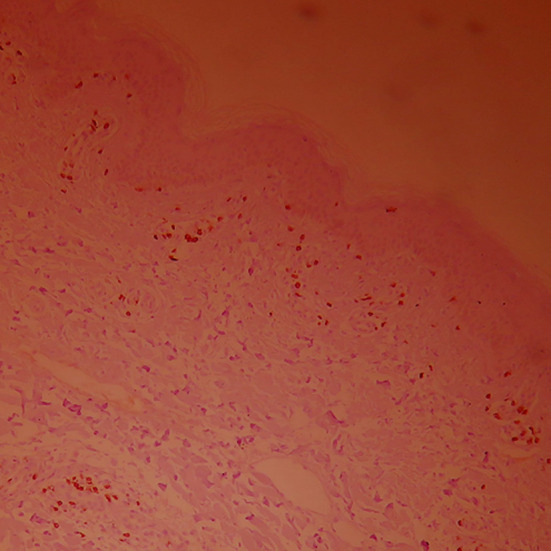
CD_4_^+^T lymphocyte expression in skin damage tissue after combined treatment (×100).

T lymphocyte subpopulation expression level in damaged skin (Fig.4-6) The manifestation of positive CD_4_ and CD_8_T lymphocyte is brown yellow cytomembrane. After treatment, CD_4_^+^ and CD_8_^+^ T lymphocytes of both groups slightly declined, and CD_4_^+^/CD_8_^+^ slightly decreased. The comparison of both groups before and after treatment had no statistical significance (*p*>0.05, shown in Table-III). CD_4_^+^ and CD_8_^+^ T lymphocyte expression level of vitiligo patients increased, compared with normal control group (*p*<0.05), and CD_8_^+^ T lymphocyte expression level of vitiligo patients increased, compared with normal control group (*p*<0.05, shown in Table-IV).

**Table III:** CD_4_^+^ and CD_8_^+^ T lymphocyte expression level of combined group and medicine group in the skin damage part before and after treatment (x̅ ±s).

Group	n	CD_4_^+^	CD_8_^+^	CD_4_^+^/CD_8_^+^
Combined group	11			
Before treatment		11.97±6.09	8.75±4.84	1.57±0.61
Treatment for 6 months		10.03±3.99	8.27±4.52	1.46±0.56
p		0.131	0.676	0.452
Medicine group	10			
Before treatment		11.57±4.85	9.67±4.73	1.59±0.67
Treatment for 6 months		9.27±5.18	8.70±4.56	1.41±0.90
p		0.081	0.423	0.394

***Note:*** comparison of two vitiligo groups before and after treatment, P>0.05.

**Table IV:** CD_4_^+^ and CD_8_^+^ T lymphocyte expression level in the skin damage part of vitiligo patients and normal control group (x̅ ±s).

Item	n	CD_4_^+^	CD_8_^+^	CD_4_^+^/CD_8_^+^
Vitiligo patients	21	11.78±5.49	8.73±4.67	1.58±0.64
Control group	11	8.30±4.98	5.49±3.33	2.00±0.65
p		0.003	0.001	0.003

***Note:*** comparison of CD4^+^, CD8^+^ and CD4^+^/CD8^+^ between vitiligo patients and normal control group,P <0.05

## DISCUSSION

In recent years, comprehensive treatment of vitiligo has been recognized by people from all walks of life. The treatment method of oral administration and external use of drugs combined with phototherapy is often regarded as the clinical treatment method. The main compositions of compound glycyrrhizin include glycyrrhizic acid extracted from liquorice, glycine and cysteine which have the effect of anti-inflammation, anti-allergic reaction and immunoregulation as well as the effect of parahormone.^6^-^8^ Compound glycyrrhizin can promote immunoregulation, regulate T cell activation, induce interferon generation, activate NK cells, enhance T lymphocyte differentiation capacity, reduce melanocyte damage, recover melanocyte function, and recover normal skin color.^9^,^10^

Fractional laser can gasify and peel epidermis and dermis, with small slight thermal injury area. Besides, it can promote skin healing, greatly relieve patients’ pains and shorten treatment time.^11^,^12^ Some researchers have found that, fractional laser combined with multiple therapies can achieve the better effect on persistent vitiligo treatment.^13^-^15^ The principle of vitiligo treatment by fractional laser is as follows: it stimulates the skin damage area to secrete the inflammatory cytokine so as to promote melanocyte proliferation and migration, construct drug delivery path and facilitate external drug absorption. Fractional laser can form microscopic pores consistent with the light beam in the therapy area. The fine pore diameter is beneficial to drug delivery. After fractional laser treatment, triamcinolone acetonide injection is encapsulated immediately, which can significantly improve the curative effect and stimulate percutaneous immune induction

T lymphocyte as a major regulating cell and effector cell can recognize and dispose antigen, generate multiple cytokines, maintain steady state of system and balance immunity. The laboratory investigation showed that, after CD4^+^ T lymphocytes in the mice were removed, CD3^+^ T lymphocytes increased, and more than a half of mice showed the features of vitiligo.^16^,^17^ The Chinese investigator Jiang Yu found that the immunity of patients with generalized vitiligo was low, CD4^+^ T lymphocyte declined, and CD8^+^ T lymphocyte rose. After the immunomodulatory was used, CD4^+^ T lymphocyte rose, and CD8^+^ T lymphocyte declined to different degree. Besides, patients’ immunity improved.^18^,^19^

### Limitations of the study:

The sample size should be expanded to further study whether the effective therapeutic mechanism of compound glycyrrhizin combined with fractional laser and triamcinolone acetonide injection in vitiligo treatment is related to T lymphocyte subpopulation regulation.

The reports on the detection of peripheral blood T lymphocyte subpopulation of vitiligo patients are different, and most studies hold that CD4^+^ T lymphocyte declines, CD8^+^ T lymphocyte rises and CD4^+^/ CD8^+^ lowers. This study found that, CD4^+^ T lymphocyte of vitiligo patients declined; CD4^+^/ CD8^+^ lowered, compared with the normal control group; CD8^+^ T lymphocyte rise was not found. This might be related to vitiligo vulgaris in the stable phase. Some studies believed that, T lymphocyte subpopulation of vitiligo patients in the progressive phase changes more obviously than normal people.^20^ In the damaged skin, CD4^+^T and CD8^+^T lymphocytes of vitiligo patients rose, compared with the normal control group, and T lymphocyte migration might exist in blood and damaged skin of vitiligo patients.

## CONCLUSIONS

Compound glycyrrhizin combined with fractional laser and triamcinolone acetonide injection in vitiligo treatment showed the definite curative effect. After treatment, T lymphocyte subpopulation changes in serum and damaged skin had no statistical significance. Thus, it is inferred that the effective mechanism of combined and independent treatment of vitiligo in the stable phase may have no obvious relation with T lymphocyte subpopulation.

### Authors’ Contributions:

**LL:** Designed this study and prepared this manuscript, and is responsible and accountable for the accuracy or integrity of the work.

**LG & YZ:** Collected and analyzed clinical data.

***Note:*** Ling Li and Lei Gao contribute to this work equally.
